# 

**Published:** 1906-05-12

**Authors:** 


### BOOKS RECEIVED.

Baillière, Tindall, and Cox.

"Philosophy of Voice." 10th Edition. By Chas. Lunn.

John Bale, Sons and Danielsson, Limited.

"Modern Surgical Technique." By C. Y. Pearson, M.D.

John Murray.

"The Morphology of Normal and Pathological Blood." By G. A. Buckmaster, M.D.

J. and A. Churchill.

"On Physical Training in Schools." By W. P. Herringham, M.D.

"Heath's Minor Surgery and Bandaging." 13th Edition. By Bilton Pollard, F.R.C.S.

Eliot Boothroyd.

"Low's Handbook to the Charities of London, 1906."

Masson et Cie., Paris.

"Eléments d'Electrothérapie Clinique." By A. Zimmern.

### Medical Appointments and Vacancies.

### VACANCIES.

Birmingham and Midland Eye Hospital.—Resident Surgical Officer.

Birmingham and Midland Hospital for Skin and Urinary Diseases, John Bright Street, Birmingham.—Clinical Assistant.

Brecknock County and Borough Infirmary.—Resident House Surgeon.

Buxton, Derbyshire, Devonshire Hospital.—Assistant House Surgeon.

Devon County Asylum.—Third Assistant Medical Officer.

Dudley, Guest Hospital.—Assistant House Surgeon.

Hartshill, Stoke-on-Trent, North Staffordshire Infirmary and Eye Hospital.—Junior House Surgeon.

Macclesfield General Infirmary.—Junior House Surgeon.

Maidstone, West Kent General Hospital.—Senior House Surgeon.

Manchester and Salford Hospital for Skin Diseases.—House Surgeon.

National Dental Hospital and College, Great Portland Street, W.—Anæsthetist.

Royal Free Hospital, Gray's Inn Road, W.C.—Assistant to the Clinical Pathologist; also Junior Obstetric Assistant and Assistant Anæsthetist. All females.

Sheffield, Royal Infirmary.—Junior Assistant House Surgeon. Also Junior House Physician.

Surrey Dispensary, Southwark, S.E.—Resident Medical Officer.

Swansea General and Eye Hospital.—House Surgeon.

Taunton and Somerset Hospital, Taunton.—Resident Assistant House Surgeon.

Victoria Hospital for Children, Tite Street, Chelsea, S.W.—Honorary Anæsthetist. Also Surgeon to In-patients. Also House Surgeon.

Wells, Somerset and Bath Asylum.—Second Assistant Medical Officer.

West End Hospital for Diseases of the Nervous System, 73 Welbeck Street, W.—Clinical Assistant.

Wigan, Royal Albert Edward Infirmary and Dispensary.—Junior House Surgeon.

"Lloyd's News" is sending out gratis and post free, to all who apply for it, a free book of 120 pages of excellent reading matter. This free book is profusely illustrated, and tells the story of the famous international library, the monumental work in twenty volumes edited by the late Dr. Richard Garnett, and now being offered at such a temptingly low price. Further particulars appear on page iii.

### NOTICE TO CORRESPONDENTS.

**All MSS., Letters, Books for Review, and other matters intended for the Editor should be addressed The Editor, "The Hospital," The Hospital Buildings, 28 & 29 Southampton Street, Strand, W.C.**

**The Editor cannot undertake to return rejected MS., even when accompanied by stamped directed envelope.**

### Notice to Advertisers and Subscribers.

**All Advertisements, Orders for Copies of The Hospital, and Business Communications should be addressed to THE MANAGER (Not to the Editor), The Hospital, 28 & 29 Southampton Street, Strand London, W.C.**

**The Cost of Subscription, post free, is as follows:—Per week, 3½d.; per quarter, 3s. 3d.; per half-year, 6s. 6d.; per annum, 13s. Foreign and Colonial:—Per annum, 19s. 6d., payable, in all cases, in advance.**

**NOTICE.—Vol. XXXIX. of "The Hospital" (October 1905 to March 1906), in handsome cloth binding, will shortly be on sale at the office of this Journal, price 10s. 6d. Cases for binding the Numbers contained in this Volume 2s. Index to Vol. XXXIX., 3½d., post free.**

**The Monthly Part for May is now ready. Price 1s., by post, 1s. 3d.**

